# NF-κB/OCT−1 Mediated Upregulation of CXCL14 Chemokine Mobilizes Mucosal Effector Memory CD44+CD62L-CD4+ and CD8+ TEM Cells, and NK Cells Associated with Protection Against Genital Herpes

**DOI:** 10.20411/pai.v11i1.879

**Published:** 2026-06-03

**Authors:** Yassir Lekbach, Swayam Prakash, Hawa Vahed, Afshana Quadiri, Azizur Rahman, El Houcine El Fatimi, Joshua Christian Dorotta, Baverly Sabathini Suoth, Chhaya Maurya, America Garcia, Gina Park, Lbachir BenMohamed

**Affiliations:** 1 Laboratory of Cellular and Molecular Immunology, Gavin Herbert Eye Institute, University of California, Irvine, School of Medicine, Irvine, California; 2 Institute for Immunology, University of California, Irvine, School of Medicine, Irvine, California; 3 Department of Vaccines and Immunotherapies, TechImmune, LLC, University Lab Partners, Irvine, California

**Keywords:** HSV-2, Genital Herpes, CXCL14, Mucosa, Memory CD8^+^ T Cells, NF-κB

## Abstract

**Background:**

Mucosal chemokines (eg, CCL25, CCL28, CXCL14, and CXCL17) play key roles in protecting mucosal surfaces against invading infectious pathogens. However, their specific contributions to protection against genital herpes remain to be fully elucidated. Here, we investigated the role of CXCL14 as a mediator of mucosal immunity against genital HSV-2 infection and disease.

**Methods:**

*CXCL14* expression was analyzed in HSV-specific CD8^+^ T cells from HSV-2–infected asymptomatic and symptomatic women, in primary human vaginal epithelial cells, and in a murine genital HSV-2 infection model. *CXCL14(–/–)* deficient mice and wild-type (WT) mice were compared for genital disease severity, vaginal viral loads, survival, immune cell recruitment, and T-cell effector function following intravaginal HSV-2 infection. Chromatin immunoprecipitation assays were performed to identify transcription factors binding to the *CXCL14* promoter after HSV-2 infection.

**Results:**

CXCL14 was homeostatically expressed at the genital tract mucosal surface, and HSV-specific CD8^+^ T cells from HSV-2–infected asymptomatic women expressed significantly higher levels of CXCL14 than those from symptomatic women. HSV-2 infection rapidly induced *CXCL14* transcription and production in primary human vaginal epithelial cells and in murine vaginal mucocutaneous tissue. *CXCL14(–/–)* mice developed more severe genital lesions, higher vaginal viral loads, and lower survival compared to WT mice. In addition, *CXCL14* deficiency impaired the recruitment of natural killer (NK) cells, neutrophils, and CD44^+^CD62L^–^ effector memory CD4^+^ and CD8^+^ T cells to the infected vaginal mucosa. It reduced T cell effector functions, including production of IFN-γ, TNF-α, and Granzyme B. Mechanistically, NF-κB and OCT-1 transcription factors bound to the CXCL14 promoter within hours of HSV-2 infection.

**Conclusions:**

Our findings demonstrate that NF-κB- and OCT-1–driven CXCL14 expression is crucial for orchestrating early innate and T-cell responses that protect against genital HSV-2 infection and disease. These results suggest that CXCL14 is an important immunoregulatory chemokine triggered early after epithelial viral infection, facilitating the induction of effective mucosal protective immunity against genital herpes.

## INTRODUCTION

Genital herpes is among the most common sexually transmitted infections, caused by two related viruses: herpes simplex virus type 1 (HSV-1) and herpes simplex virus type 2 (HSV-2). HSV-2 accounts for the majority of recurrent and symptomatic genital infections [1]. Global estimates indicate that approximately 520 million individuals aged 15–49 years (13% of this age group) are infected with HSV-2, with women disproportionately affected due to the higher efficiency of male-to-female transmission [2]. In 2020, around 205 million people in the same age range (5.3%) experienced at least one symptomatic episode of genital herpes, with 92% of those cases attributed to HSV-2 [2]. Moreover, approximately 40–60 million individuals are infected with HSV-2 in the United States alone, with nearly 600,000 to 800,000 reported annual clinical cases [3–8].

HSV-2 is a neurotropic virus classified under the *Alpha Herpesviridae* subfamily. Following replication in mucosal epithelial tissues, HSV-2 establishes lifelong latency in sensory neurons of the dorsal root ganglia (DRG), from which it can periodically reactivate [4, 9–11]. HSV-2 frequently undergoes asymptomatic reactivation, occasionally resulting in detectable lesions at genital sites [12]. These reactivation events are primarily controlled by the host immune response, which restricts their frequency and duration [13]. Multiple cell types contribute to the containment and clearance of HSV-2, including keratinocytes and epithelial cells that secrete chemokines to recruit T cells to the site of infection, infiltrating CD4^+^ T cells that produce interferon-gamma (IFN-γ), and effector CD8^+^ T cells that mediate viral clearance through cytolytic activity and IFN-γ-dependent suppression of viral replication [14–17].

During genital HSV-2 infection, the vaginal mucosa (VM) responds by producing a variety of mucosal chemokines that coordinate the recruitment and activation of immune cells [18]. However, their specific roles in shaping mucosal immune responses during infection remain poorly defined. This is particularly important given that the VM is considered a “closed immunological compartment,” which limits the infiltration of circulating lymphocytes from draining lymph nodes [19–22]. Although local immune responses, particularly B- and T-cell activity, are essential for controlling viral replication and disease severity [24–28], the mechanisms governing their mobilization and retention within the VM remain incompletely understood. Among the chemokines implicated in mucosal immunity, 4, CXCL14, CXCL17, CCL25, and CCL28, are constitutively expressed at mucosal surfaces and help regulate homeostatic immune surveillance [23–25]. In particular, CXCL14, also known as BRAK (Breast and Kidney-Expressed Chemokine) or BMAC (BM-40-SPARC-Associated), is constitutively expressed in several tissues, including the digestive, respiratory, and reproductive tracts, as well as in various immune cells, such as activated B cells, dendritic cells (DCs), and monocytes [23, 26]. CXCL14 facilitates the recruitment of neutrophils, eosinophils, macrophages, T cells, and DCs [27–30]. It has also been identified as a potential biomarker for several diseases, including cardiac dysfunction [31]. Witte et al. showed that CXCL14 can directly interact with CXCR4 to mediate platelet function and migration [32]. Additionally, CXCL14 contributes to the early antimicrobial defense against bacteria, fungi, and certain viruses [33, 34]. Although CXCL14 has been reported to interact with CXCR4 and CXCR7 [35], the exact receptor mediating its immunological functions remains unclear, and its role in genital mucosal immunity, particularly in the context of HSV-2 infection, has yet to be elucidated.

In this study, we first analyzed bulk RNA sequencing data from our previous investigation of HSV-specific CD8^+^ T cells to identify differential regulation of chemokine pathways in symptomatic (SYMP) and asymptomatic (ASYMP) women following HSV infection. Subsequently, we identified CXCL14 as being highly expressed in HSV-infected ASYMP women. Moreover, using the *CXCL14* knockout mouse model (*CXCL14(–/–)*), we confirmed a protective role for the CXCL14 chemokine axis against genital herpes infection and disease. CXCL14 chemokine-mediated protection against genital herpes is associated with the mobilization of antiviral effector memory CD44^+^CD62L-CD4^+^ T_EM_ cells, CD44^+^CD62L-CD8^+^ T_EM_ cells, neutrophils, and natural killer (NK) cells into the VM of HSV-2-infected mice. We discuss the potential use of the mucosal chemokine CXCL14 in a prime/pull/keep (PPK) therapeutic vaccine to enhance genital herpes T-cell immunity and protect against disease caused by HSV-2, as well as potentially other sexually transmitted viruses.

## RESULTS

### CXCL14 Expression is Elevated in ASYMP Compared to SYMP HSV-2-Infected Women

**Figure 1.**
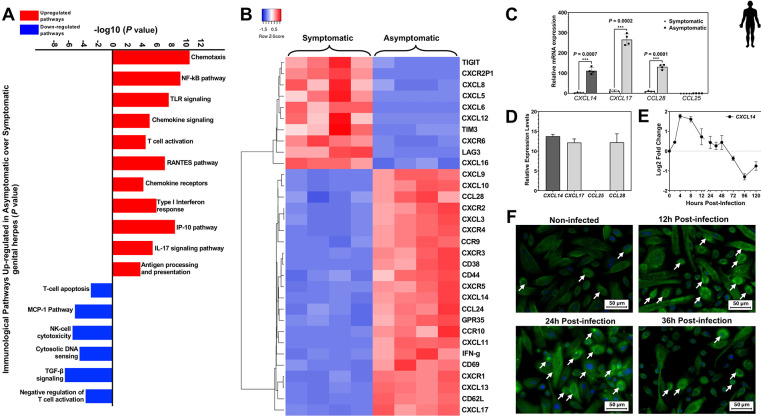
**CXCL14, CXCL17, and CCL28 expression levels in CD8+ T cells from PBMCs of herpesinfected SYMP compared to ASYMP patients.** (A) Major immunological pathways found to be differentially expressed among ASYMP patients in comparison to SYMP patients. (B) Heatmap of selected chemokines and immune-related genes showing distinct expression profiles in SYMP vs ASYMP genital herpes patients. Data are presented as row Z-scores from RNA-seq analysis (GSE241702). (C) CXCL14, CXCL17, and CCL28 mRNA expression levels in SYMP versus ASYMP genital herpes patients. ASYMP individuals showed significantly higher expression of these chemokines (*P* < 0.05). (D) Relative baseline expression levels of *CXCL14, CXCL17, CCL25*, and *CCL28* genes in human vaginal epithelial cells (VECs) at homeostasis. CCL25 expression was not detected in these cells. (E) Time-course analysis of CXCL14 mRNA expression in HSV-2-infected human primary VECs. CXCL14 is upregulated at early time points (4-24 hours) and downregulated thereafter. (F) Immunofluorescence images of human primary VECs showing CXCL14 localization (green) at various time points following HSV-2 infection (12, 24, and 36 hours p.i.). Arrows indicate cells with a strong CXCL14 signal. Nuclei were counterstained with DAPI (blue). Data are presented as mean ± SD from three independent experiments. Statistical significance was determined using an unpaired *t-*test.

We first examined whether mucosal chemokine pathways differ between HSV-specific CD8^+^ T cells from SYMP and ASYMP HSV-2-infected women. The major immunological pathways that we found to be significantly upregulated among ASYMP herpes participants were the Chemotaxis pathway, NF-kB pathway, TLR signaling pathway, RANTES pathway, Chemokine receptor pathway, IP-10 pathway, IL-17 pathway, and antigen processing and presentation pathway (Figure 1A). Whereas pathways found to be downregulated in ASYMP herpes participants included T-cell apoptosis, MCP-1, NK-cell cytotoxicity, TGF-β signaling, and negative regulation of T-cell activation pathways (Figure 1A). All these pathways were statistically significant, with *P-*values < 0.0001. Using RNA-seq data from the publicly available dataset GSE241702, we generated a heatmap of differentially expressed chemokine-related genes (Figure 1B). Several chemokines, including CXCL14, CXCL17, and CCL28, showed markedly higher expression in ASYMP patients than in SYMP patients (Figure 1C). Thus, the heatmap of row Z-scores from RNA-seq analysis (GSE241702) shows a clear difference in the mRNA expression levels of the mucosal chemokines CXCL14, CXCL17, and CCL28 in SYMP vs ASYMP patients with genital herpes. In addition to chemokines, the heatmap also showed differences in T-cell activation and inhibitory receptor markers. ASYMP patients demonstrated higher expression of markers associated with sustained T-cell function and tissue retention (eg, CD44 and CD69) and chemokine receptors (eg, CXCR3, CXCR4, and CCR9), which support trafficking to mucosal tissues. SYMP patients, by contrast, showed higher expression of inhibitory receptors, including TIGIT, TIM-3 (HAVCR2), and LAG-3, as well as upregulation of genes associated with immune exhaustion. These patterns suggest that ASYMP CD8^+^ T cells may retain a more functional, tissue-resident phenotype, whereas SYMP cells may be skewed toward an exhausted state.

### CXCL14 is Highly Induced in Primary Human Vaginal Epithelial Cells Following HSV-2 Infection

We next examined mucosal chemokine expression in primary human vaginal epithelial cells (VECs). Under homeostatic conditions, *CXCL14*, *CXCL17*, and *CCL28* mRNA were detected, while *CCL25* was not (Figure 1D). Next, we infected cells with the HSV-2 strain MS at a multiplicity of infection (MOI) of 10. Phase-contrast microscopy revealed progressive cytopathic effects, characterized by cell rounding, detachment, and lysis, which began at 24 hours post-infection (p.i.) and became more severe by 72 hours p.i. (Supplementary Figure 1A). These changes were accompanied by a steady increase in HSV-2 genomic DNA levels, confirming productive infection in these cells (Supplementary Figure 1B).

The MOI of 10 used to infect human VECs was selected to achieve a synchronous, uniformly infected monolayer and to ensure robust early viral gene expression and downstream host-response readouts within the defined experimental window. Regarding the comparison with the mouse infection dose, MOI (in vitro) and plaque-forming unit (PFU) delivered per animal (in vivo) are different units and cannot be converted directly. However, both conditions were chosen to reliably generate productive infection and measurable host responses in each model. For example, previous studies have infected the same primary human VECs (ATCC PCS-480-010) used in this study with HSV-2 strain G at comparable high MOIs to ensure robust epithelial infection and downstream host-response analyses [36]. Further, the morphology of cytopathic effects in monolayers (cell rounding, detachment, and lysis) will not resemble that in infected tissues, where epithelial architecture and extracellular matrix constrain cell detachment and where pathology may present as focal epithelial disruption/sloughing and inflammatory changes rather than widespread monolayer collapse. The observed cytopathic effect (CPE) confirms productive infection in vitro but is not intended to recapitulate tissue histomorphology.

Importantly, we found that *CXCL14* mRNA expression was strongly induced in response to infection, with mRNA levels peaking between 4 hours and 24 hours p.i., followed by a decline (Figure 1E). *CXCL14* protein expression was also detected in primary human VECs, peaking at 24 hours p.i. (Figure 1F). Altogether, these results indicate that CXCL14 is an early-response chemokine that is rapidly upregulated in primary human VECs after HSV-2 infection and may contribute to the early antiviral response.

### CXCL14 is Highly Produced in the VM of HSV-2-Infected B6 Mice

**Figure 2.**
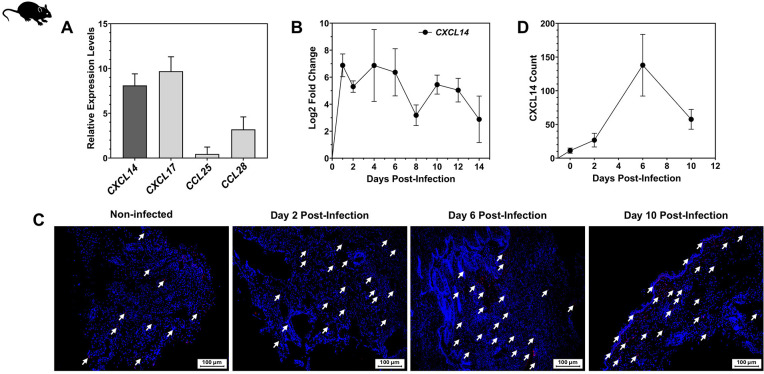
**CXCL14 expression kinetics in mice following HSV-2 infection.** (A) Relative expression levels of CXCL14, CXCL17, CCL25, and CCL28 mRNA in mice vaginal tissues at homeostasis, as measured by RT-qPCR. (B) Log₂ fold change in CXCL14 expression over time post-HSV-2 infection, showing a peak in early infection (day 2–4) followed by a gradual decline. (C) Representative immunofluorescence images showing CXCL14 protein expression (red arrows) in vaginal tissue sections from non-infected mice and from HSV-2-infected mice at days 2, 6, and 10 post-infection (p.i.). Nuclei are counterstained with DAPI (blue). (D) Quantification of CXCL14 proteins in non-infected tissues (0 day) and infected tissues at days 2, 6, and 10 p.i. Quantitative analysis was performed using ImageJ software. Data are presented as mean ± SD from three independent experiments.

We examined the expression of mucosal chemokines in the VM of C57BL/6 (B6) mice using RT-qPCR. Under homeostatic conditions, all 4 mucosal chemokines (*CXCL14*, *CXCL17*, *CCL25*, and *CCL28*) were expressed in the VM (Figure 2A). However, *CCL25* was expressed at very low levels. Following intravaginal infection with 5 × 10^5^ PFU of HSV-2 strain MS, we observed a significant upregulation of *CXCL14* mRNA as early as day 1 p.i., peaking between days 2 and 4 and then gradually declining over time (Figure 2B). Next, we assessed CXCL14 protein expression in VM tissues at baseline and at 2, 6, and 10 days p.i. following HSV-2 infection. As shown in the immunofluorescent (IF) images (Figure 2C), CXCL14 was detectable under homeostatic conditions and increased following infection, peaking around day 6 p.i.

### CXCL14 Deficiency Exacerbates Genital Herpes Severity and Increases Viral Replication

**Figure 3.**
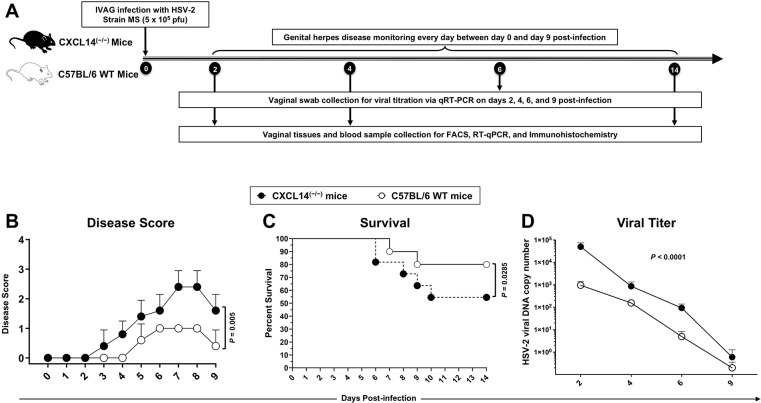
**Genital herpes disease progression and survival in *CXCL14*^*(–/–)*^ and WT B6 mice following HSV-2 infection.** (A) Experimental design: *CXCL14(–/–)* mice (n = 10) and WT mice (n = 10) were infected intravaginally with 5 × 10^5^ PFU of HSV-2 strain MS. *CXCL14(–/–)* and WT mice were scored daily for 9 days p.i. for symptoms and severity of genital herpes, as described in Materials and Methods. Vaginal swabs were collected on days 2, 4, 6, and 9 post infection (p.i.) to quantify viral load by quantitative PCR (qPCR). On day 14 p.i., mice were euthanized for gene expression analysis (qPCR on vaginal mucosa (VM) tissue from days 1–14), immunohistochemistry (VM on days 1–14), and flow cytometry (PBMCs on day 14). (B) cumulative scoring of vaginal lesions observed during infection, The severity of genital herpetic lesions was scored on a scale of 0–4, where 0 = no disease, 1 = swelling of external vagina; 2 = swelling and redness of external vagina, 3 = severe swelling and redness of vagina and surrounding tissue and hair loss in the genital area, 4 = ulceration and hair loss in the genital and surrounding tissue. (C) Survival rates of *CXCL14(–/–)* and WT mice up to day 14 p.i. By the end of the experiment, 4 out of the *CXCL14(–/–)* mice had died, while only 2 WT mice did not survive. (D) Vaginal viral genomic DNA levels for 9 days p.i. Measured by qPCR.

To further evaluate the role of CXCL14 in protection against genital herpes, we compared infection and disease outcomes between *CXCL14(–/–)* and WT mice. Both groups (n = 10) were infected intravaginally on day 0 with 5 × 10^5^ PFU of HSV-2 strain MS (Figure 3A). We monitored mice daily for 14 days for survival and genital disease severity, as described in the Materials and Methods section. We collected vaginal swabs on days 2, 4, 6, and 9 p.i. to measure HSV-2 genomic DNA levels using quantitative PCR (qPCR). As shown in Figure 3B, *CXCL14(–/–)* mice developed significantly more severe genital lesions by day 9 compared to WT controls. Survival analysis also revealed a significant difference between the two groups, with higher mortality in knockout mice (Figure 3C). Furthermore, viral genomic DNA levels were consistently higher in the VM of infected *CXCL14(–/–)* mice (Figure 3D), indicating impaired viral control in the absence of CXCL14.

### CXCL14 Deficiency Reduces Innate and Adaptive Immune Cell Infiltration in the VM

Given the association between CXCL14 deficiency and increased HSV-2 disease severity and viral replication (Figure 3), we next investigated whether CXCL14 deficiency also alters immune cell infiltration in the VM. *CXCL14(–/–)* and WT mice (n = 10) were intravaginally infected on day 0 with 5 × 10^5^ PFU of HSV-2 strain MS.

**Figure 4.**
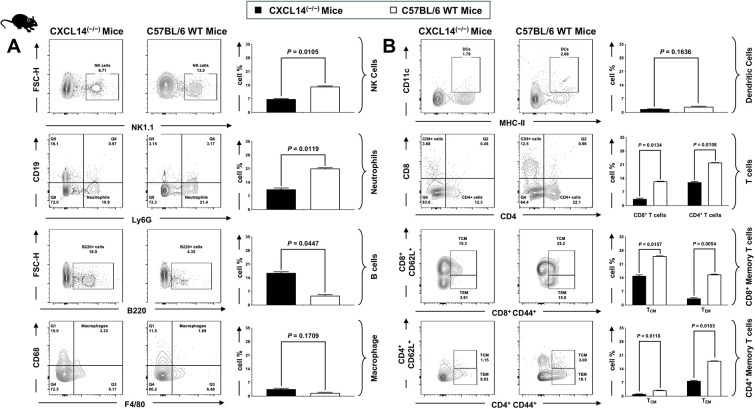
**Loss of CXCL14 alters the composition of innate and adaptive immune cells in the genital tract.** Flow cytometry analysis comparing immune cell populations in the genital tracts of CXCL14(–/–) and C57BL/6 WT mice. (A) (Left panels) Representative contour plots and quantification of innate immune cells, including NK cells (NK1.1^+^), neutrophils (CD19^–^Ly6G^+^), B cells (B220^+^), and macrophages (CD68^+^F4/80^+^). *CXCL14(–/–)* mice exhibit significantly lower frequencies of NK cells and neutrophils compared to WT mice. No significant differences were observed in macrophage populations, while B cells were significantly higher in *CXCL14(–/–)* mice. (B) (Right panels) Representative flow cytometry plots and quantification of dendritic cells (CD11c^+^MHC-II^+^), total T cells (CD8^+^ and CD4^+^), and memory T cell subsets (CD8^+^CD44^+^ and CD4^+^CD44^+^), including central memory (TCM: CD62L^+^) and effector memory (TEM: CD62L^–^) cells. CXCL14(–/–) mice show a significant reduction in CD8^+^ and CD4^+^ T cells, as well as both TCM and TEM subsets, compared to WT controls. Bar graphs represent mean ± SEM, n = 10 mice/group. An unpaired *t-*test assessed statistical significance. *P-*values are indicated on graphs.

On day 14 p.i., we euthanized the mice and processed the VM tissues into single-cell suspensions for flow cytometric analysis of various immune cell populations. Compared to WT controls, *CXCL14(–/–)* mice showed significantly lower frequencies of NK cells (*P* = 0.0105), neutrophils (*P* = 0.0119), CD8^+^ T cells (*P* = 0.0134), and CD4^+^ T cells (*P* = 0.0158) (Figure 4A). Interestingly, B cell frequencies were significantly elevated in *CXCL14(–/–)* mice (*P* = 0.0158). In contrast, macrophage (*P* = 0.1709) and DC (*P* = 0.1636) levels remained unchanged. Further analysis showed a reduction in both CD4^+^ and CD8^+^ effector memory T (T_EM_) cells (CD44^+^CD62L^–^) and CD4^+^ and CD8^+^ central memory T (T_CM_) cells (CD44^+^CD62L^+^) in the VM of *CXCL14(–/–)* mice relative to WT (Figure 4B). These findings demonstrate that CXCL14 is essential for the efficient recruitment of both innate and adaptive immune cells to the VM following HSV-2 infection.

### CXCL14 Deficiency Impairs CD4^+^ and CD8^+^ T-cell Effector Function

**Figure 5.**
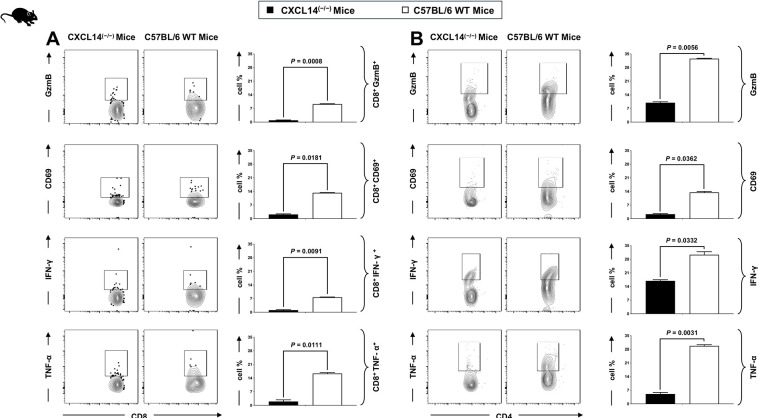
**CXCL14 deficiency impairs activation and effector function of HSV-specific CD8+ and CD4+ T cells in the genital tract.** Flow cytometry analysis of genital tract-infiltrating CD8^+^ and CD4^+^ T cells from CXCL14(–/–) and C57BL/6 WT mice following HSV-2 infection. (A) (Left panels) Representative contour plots and quantification of CD8^+^ T cells expressing Granzyme B, CD69, IFN-γ, and TNF-α. (B) (Right panels) Representative plots and quantification of CD4^+^ T cells expressing the same activation and effector markers. *CXCL14(–/–)* mice exhibited significantly reduced frequencies of both CD8^+^ and CD4^+^ T cells expressing Granzyme B, CD69, IFN-γ, and TNF-α compared to WT mice, indicating impaired antiviral T cell responses in the absence of CXCL14. Bar graphs show mean ± SD. n = 10 mice/group. Statistical analysis was performed using an unpaired *t-*test, and *P-*values are indicated.

To determine whether CXCL14 deficiency influences the functional capacity of T cells in infected tissues, we compared CD4^+^ and CD8^+^ T cell responses in *CXCL14(–/–)* and WT mice. Both groups (n = 10) were intravaginally infected with 5 × 10^5^ PFU of HSV-2 strain MS. On day 14 p.i., we isolated single-cell suspensions from infected VMs and analyzed them by flow cytometry. In *CXCL14(–/–)* mice, we observed a reduction in the frequencies of CD4^+^ and CD8^+^ T cells that produce Granzyme B, IFN-γ, and TNF-α, compared with WT controls (Figures 5A and 5B). The percentage of CD69^+^ cells in both T cell subsets was also lower in knockout mice, indicating reduced local activation or retention in the VM. In conclusion, these results suggest that CXCL14 is necessary for the optimal effector function of CD4^+^ and CD8^+^ T cells in the infected VM.

### CXCL14 Deficiency Impairs CD4^+^ and CD8^+^ T-Cell Recruitment to Infected VM

**Figure 6.**
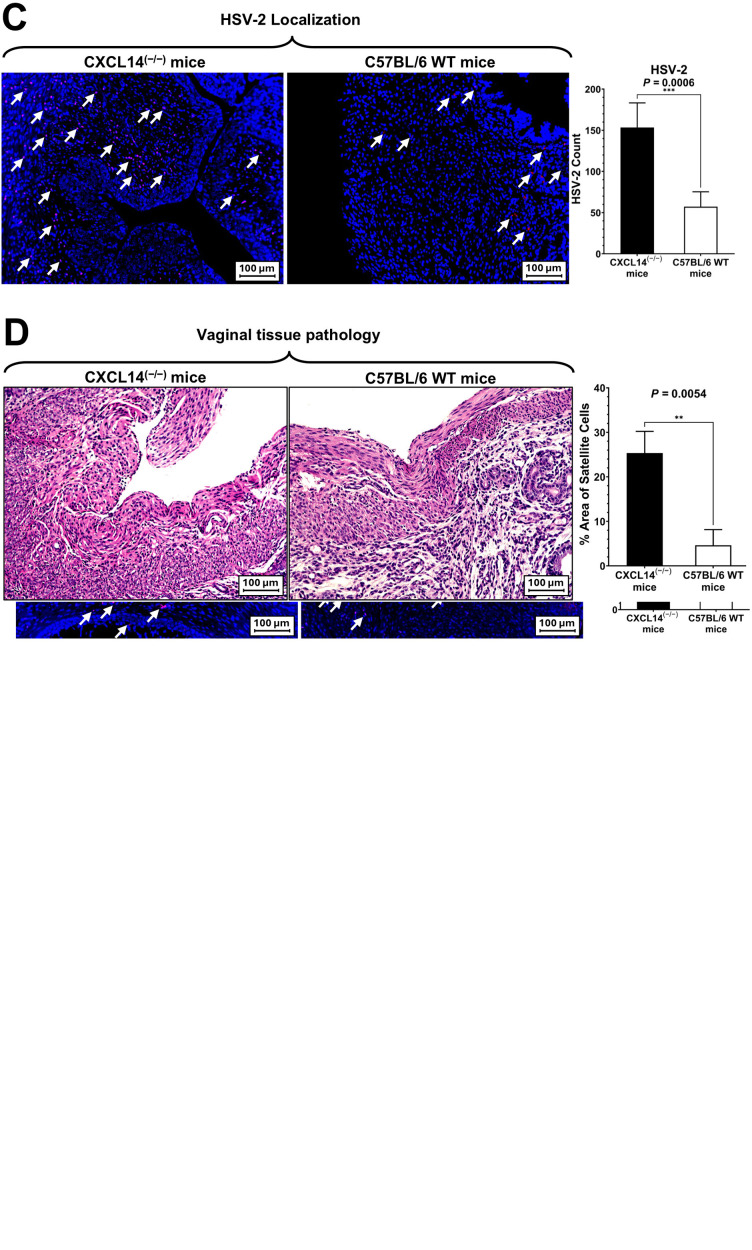
**CXCL14 deficiency impairs T cell infiltration and enhances HSV-2 localization and pathology in the vaginal tissue.** (A) Representative immunofluorescence images showing infiltration of CD4^+^ T cells (green, indicated by arrows) in vaginal tissue of *CXCL14(–/–)* and C57BL/6 WT mice. Nuclei are stained with DAPI (blue). Quantification of CD4^+^ T cell infiltration is shown in the adjacent bar graph. (B) Representative immunofluorescence images of CD8^+^ T cell infiltration (red, indicated by arrows) in vaginal tissues of both groups, with corresponding quantification. (C) HSV-2 localization in vaginal tissues was detected by immunofluorescence. *CXCL14(–/–)* mice exhibited significantly higher HSV-2 levels (red) than WT controls. (D) Hematoxylin and eosin (H&E) staining of vaginal tissues reveals increased epithelial disruption and inflammation in *CXCL14(–/–)* mice compared to WT. All images are shown at a 100 µm scale. All quantitative analyses were performed using ImageJ software. Bar graphs represent mean ± SD, n = 10 mice/group. Statistical significance was determined using an unpaired *t-*test (*P* < 0.05).

To evaluate whether CXCL14 contributes to T cell recruitment during infection, we compared T cell infiltration in *CXCL14(–/–)* and WT mice following intravaginal infection with HSV-2 strain MS (*n* = 10 per group). We collected VM tissues on day 14 p.i. and analyzed them by IF. Staining for CD4 and CD8 revealed a marked reduction in both T-cell populations in CXCL14(–/–) mice compared to WT controls (*P* < 0.05; Figures 6A and 6B). Consistent with this, HSV antigen staining showed significantly higher viral loads in the VM of knockout mice (*P* < 0.05; Figure 6C). Histological examination using H&E staining further demonstrated more extensive epithelial damage and inflammation in the absence of CXCL14 (Figure 6D and Supplementary Figure 2A-2B). These results indicate that CXCL14 is critical for promoting T-cell infiltration at the site of infection and for limiting tissue damage and viral burden.

### CXCL14 Deficiency Alters the Expression of Other Mucosal Chemokines During Infection

**Figure 7.**
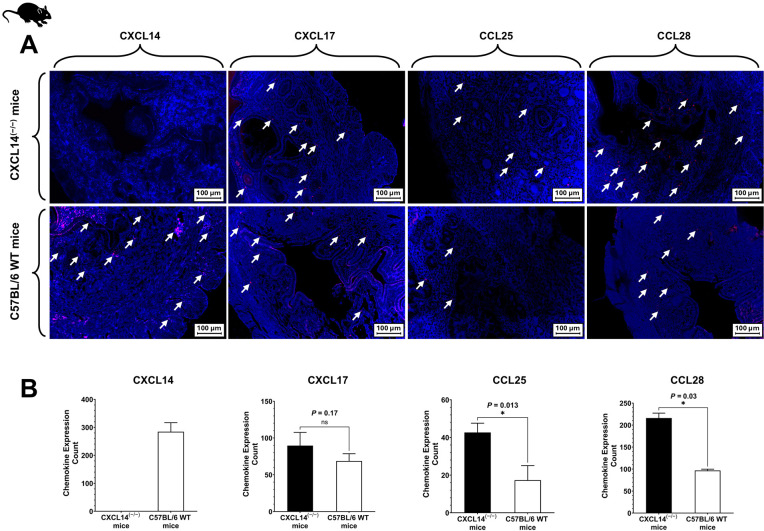
**Expression of mucosal chemokines in vaginal tissue of HSV-2-infected CXCL14(–/–) and C57BL/6 WT mice.** (A) Representative immunofluorescence images showing expression of chemokines CXCL14, CXCL17, CCL25, and CCL28 (red) in vaginal tissues of *CXCL14(–/–)* and WT mice following HSV-2 infection. Nuclei were stained with DAPI (blue). (B) Quantification of chemokine expression levels in vaginal tissues. CXCL14 expression was absent in *CXCL14(–/–)* mice as expected. Expression levels of CCL25 and CCL28 were significantly elevated in *CXCL14(–/–)* mice compared to WT, while CXCL17 expression showed no statistically significant difference. Images were captured at a 100 µm scale. All quantitative analyses were performed using ImageJ software. Bar graphs represent mean ± SD of quantified fluorescence-positive cells per tissue section. n = 10 mice/group. Statistical significance was determined using an unpaired *t-*test; *P* values are indicated.

We next assessed whether CXCL14 deficiency affects the expression of other mucosal chemokines during HSV-2 infection. On day 14 p.i., we euthanized *CXCL14(–/–)* and WT mice and collected infected VM tissues, which were then stained with antibodies against mucosal chemokines. Figure 7 compares chemokine expression levels in the VM of both groups. A significant increase in CCL28 (*P* = 0.03) and CCL25 (*P* = 0.013) protein expression was observed in the *CXCL14(–/–)* group compared to WT mice. In contrast, CXCL17 levels did not differ significantly between the two groups. These findings suggest that loss of CXCL14 may lead to a compensatory upregulation of specific mucosal chemokines, potentially as part of an altered local immune response to HSV-2 infection.

### NF-κB and OCT-1 Transcription Factors Bind to the *CXCL14* Promoter After HSV-2 Infection.

**Figure 8.**
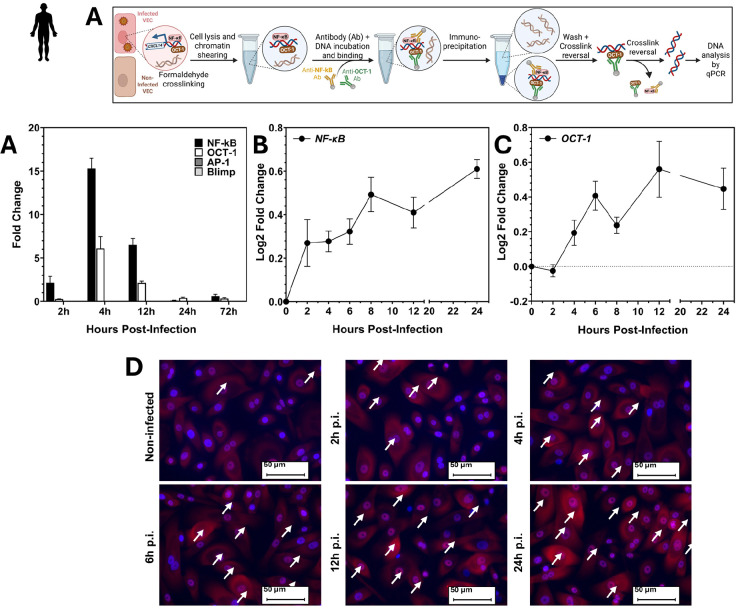
**NF-κB and OCT-1 bind the CXCL14 promoter and are activated during HSV-2 infection of VECs.** (A) ChIP experiment illustration (B) Chromatin immunoprecipitation (ChIP) analysis showing dynamic transcription factor binding (NF-κB and OCT-1) to the CXCL14 promoter at various time points after HSV-2 infection in human VECs. NF-κB binding peaked at 4 hours p.i., whereas OCT-1 showed moderate binding at earlier time points. AP-1 and Blimp-1 did not bind to the promoter region under these conditions. Data are presented as mean ± SEM from three independent experiments. Statistical significance was determined using an unpaired Student’s *t-*test where indicated. (C) and (D) NF-κB and OCT-1 mRNA levels in primary human VECs infected with HSV-2 (MOI 10), as determined by RT-qPCR. Bars show the mean ± SEM. Values are expressed as fold increases relative to non-infected cells. (E) Representative immunofluorescence images showing nuclear localization of NF-κB p65 (red) in non-infected and HSV-2–infected VECs at the indicated time points post-infection (p.i.). Nuclei are counterstained with DAPI (blue). White arrows indicate NF-κB–positive nuclei. Figure 8A was Created with BioRender.com.

We aimed to identify the transcription factors (TFs) that may bind to the promoter region of the *CXCL14* gene and drive its upregulation following HSV-2 infection. To this end, chromatin Immunoprecipitation (ChIP) assays were performed to evaluate the in vitro binding of NF-κB, OCT-1, AP-1, and Blimp-1. Chromatin was isolated from primary human VECs, both non-infected and HSV-2-infected (MOI 10), at 2, 4, 12, 24, and 72 hours. The results revealed that both NF-κB and OCT-1 bound to the *CXCL14* promoter as early as 2 hours p.i. (Figure 8B). NF-κB showed a drastic increase (∼15-fold) at 4 hours p.i., peaking early and dropping sharply thereafter. OCT-1 also showed increased binding (∼6-fold at 4 h p.i.), but to a lesser extent than NF-κB. In contrast, AP-1 and Blimp showed no detectable binding at any time point, suggesting they are not involved in regulating *CXCL14* expression under these experimental conditions.

To complement these findings, we next examined the gene expression levels of *NF-κB* and *OCT-1* in primary human VECs following HSV-2 infection. *NF-κB* expression increased steadily over the 24-hour time course (Figure 8C). *OCT-1* also displayed a gradual increase in expression, peaking between 6 hours and 8 hours p.i., then its expression was gradually elevated from 8 hours to 24 hours (Figure 8D). These results support a model in which NF-κB, and OCT-1 directly regulate *CXCL14* transcription during the early stages of HSV-2 infection. Immunofluorescence analysis further demonstrated the progressive nuclear translocation of NF-κB p65 in infected cells, starting at 2 hours p.i. and increasing through 24 hours p.i. (Figure 8E). These results support a model in which early NF-κB activation, followed by sustained OCT-1 binding, drives *CXCL14* transcription during the initial stages of HSV-2 infection.

## DISCUSSION

Our findings identify CXCL14 as a pivotal mucosal chemokine in the immune defense against genital HSV-2 infection. We demonstrated that CXCL14 is rapidly and robustly upregulated in VECs and the mouse model upon HSV-2 exposure, and that its absence leads to significantly worse infection outcomes. These results highlight CXCL14 as an integral component of the early antiviral response at the genital mucosa, orchestrating the recruitment and activation of immune cells necessary for controlling HSV-2.

Notably, we observed that HSV-2-infected ASYMP women had higher *CXCL14* expression (as well as *CXCL17* and *CCL28*) in their HSV-specific CD8^+^ T cells compared to SYMP patients. This suggests that elevated mucosal chemokine responses may contribute to effective viral containment without the development of disease symptoms. Consistent with this idea, effective local immune responses can often suppress HSV-2 replication in genital tissue before lesions form [17]. Indeed, mucosal chemokines such as CXCL14, CXCL17, CCL25, and CCL28 are regarded as crucial protectors of mucosal surfaces against pathogens [25, 37]. A recent study on genital herpes found that ASYMP HSV-2 infection was associated with heightened CCL28 levels and greater recruitment of virus-specific effector memory T cells to the VM, whereas CCL28 deficiency led to increased susceptibility to infection [37]. Similarly, our data now implicate CXCL14 as a key chemokine whose robust expression in ASYMP individuals may help mobilize protective immune cells and prevent clinical disease.

In both human vaginal epithelium and a mouse model, CXCL14 was identified as an “early responder” chemokine to HSV-2 infection. We showed that CXCL14 mRNA and protein are sharply induced within hours of viral exposure in primary VECs. This rapid upregulation positions CXCL14 among the very first wave of host factors mobilized after HSV-2 invasion. The ability of epithelial cells to secrete chemokines and cytokines immediately after sensing the virus is crucial for alerting and recruiting immune effectors [38, 39]. In vivo, CXCL14 proved essential for controlling genital HSV-2 infection. WT C57BL/6 mice responded to intravaginal HSV-2 infection with a strong induction of CXCL14 in the VM tissues. Notably, *CXCL14(–/–)* mice exhibited markedly exacerbated disease. *CXCL14(–/–)* mice developed more severe genital lesions, showed higher viral loads in vaginal tissues, and experienced greater mortality compared to WT mice. These observations provide functional evidence that CXCL14 is a protective chemokine in genital herpes. They are in line with prior reports that the loss or blockade of key chemokines can impair host control of HSV. For instance, deletion of the CCR5 receptor in mice results in poor NK cell expansion, recruitment, and activity, as well as failure to contain genital HSV-2 infection [22]. Moreover, Lima et al. demonstrated a key role of CCL5 in recruiting leukocytes into the brain of HSV-1–infected mice [40]. In the present study, we show that CXCL14 is another crucial chemokine. In the absence of CXCL14, the host immune system cannot efficiently recruit the necessary cells to the vaginal mucosal epithelium to combat the virus, resulting in uncontrolled viral replication and disease. This suggests a direct protective role for the CXCL14 chemokine axis against genital herpes infection and disease, mediated by the mobilization of protective effector memory CD44+CD62L-CD4+ and CD8+ T_EM_ cells, as well as NK cells, into the vaginal mucosal epithelium. However, one cannot exclude additive or synergistic effects of other chemokines with CXCL14 in protection against HSV-2 infection through the mobilization of immune cells. It is also possible that CXCL14 may protect against other herpesviruses (similar to HSV-2) by triggering protective mechanisms mediated by the recruitment of both specific and nonspecific T cells.

Mechanistically, CXCL14 appears to orchestrate the recruitment of a broad range of immune cells to the site of infection. *CXCL14(–/–)* mice had significantly reduced infiltration of innate immune cells (such as neutrophils and NK cells) and adaptive immune cells (CD8^+^ and CD4^+^ T lymphocytes) into the infected VM. This broad effect is consistent with known properties of CXCL14. CXCL14 is a unique homeostatic CXC chemokine that promotes immune surveillance in epithelial tissues by attracting diverse cell types, including DCs, NK cells, and T cells [26, 41, 42]. Our data support this; in the presence of CXCL14 (WT mice), large numbers of innate effectors and T cells were recruited to the VM during infection, whereas in *CXCL14(–/–)* mice, this recruitment was impaired. By day 14 p.i., CXCL14-deficient tissues showed fewer CD4^+^ and CD8^+^ T cells on immunostaining, which was dramatically corroborated by flow cytometry results. The defect in T-cell trafficking in *CXCL14(–/–)* mice was accompanied by a substantially higher local viral antigen burden and more severe tissue damage, underscoring the central role of these T cells in containing the infection. Moreover, we observed a significant decrease in the frequencies of HSV-specific effector memory CD44^+^CD62L^–^CD4^+^ T_EM_ cells and CD44^+^CD62L^–^CD8^+^ T_EM_ cells in the infected VM of *CXCL14(–/–)* mice. In the absence of CXCL14, the host appears to be unable to effectively recruit T cells (and other leukocytes) into the infected mucosa, resulting in extensive viral spread and immunopathology. This interpretation is further supported by our finding that T-cell effector functions were impaired in *CXCL14(–/–)* mice. Although the T cells that migrated into the infected vaginal tissue in knockout mice were less effective, we observed lower frequencies of CD4^+^ and CD8^+^ T cells producing key antiviral effector molecules (IFN-γ, TNF-α, and Granzyme B) than in WT mice.

Additionally, CD69 expression was reduced on T cells from CXCL14(–/–) vaginal tissues, suggesting a decrease in tissue-resident or recently activated T cells. Taken together, these results indicate that CXCL14 not only facilitates the recruitment of sufficient numbers of T cells but may also promote their activation or retention at the site of infection. The net result is a failure to mount a rapid, effective immune response to HSV-2.

Interestingly, we observed a selective increase in B cell frequencies in *CXCL14(–/–)* mice, despite decreases in most other leukocytes. *CXCL14(–/–)* mice showed greater B-cell accumulation in the infected VM than WT mice. This may reflect a compensatory or dysregulated immune response. One possible explanation is that the loss of CXCL14 and the consequent impairment in T cell and innate cell recruitment lead to poorer early viral control, resulting in higher antigen loads that drive a stronger B cell response. Additionally, our analysis of other mucosal chemokines in *CXCL14(–/–)* mice revealed significantly elevated levels of CCL28 and CCL25 protein in the infected tissue (with CXCL17 unchanged). CCL28 is a mucosa-associated chemokine that attracts IgA-secreting plasma cells and CCR10^+^ effector T cells [43–45]. The upregulation of CCL28 in *CXCL14(–/–)* mice may represent a compensatory response aimed at recruiting lymphocytes through alternative pathways. In particular, increased CCL28 levels may explain the heightened B-cell infiltration, as CCL28 can recruit memory B cells and plasma cells to mucosal sites [37]. Intriguingly, the immune system attempts to induce other chemokines in the absence of CXCL14, perhaps as a fail-safe mechanism. However, in our model, this compensation was insufficient; the *CXCL14(–/–)* mice still exhibited more severe infection. This suggests that CXCL14 has a non-redundant protective role that other chemokines cannot fully substitute. It will be worthwhile in future studies to examine if the timing, cell sources, or gradient formation of these compensatory chemokines differ such that they cannot replace CXCL14’s function in the critical early phase of infection.

Our data also shed light on the molecular regulation of CXCL14 expression during HSV-2 infection. Using a ChIP assay, we found that the transcription factors NF-κB and OCT-1 bound to the *CXCL14* promoter in human VECs within hours of HSV-2 exposure. NF-κB is a master regulator of inflammatory gene transcription, inducing numerous cytokine and chemokine genes in response to infection or signaling from pattern-recognition and cytokine receptors [46, 47]. Using ChIP, Liu et al. demonstrated that NF-κB bound to the *CXCL8* and *CXCL5* promoters in tumorigenic (1170-I) cells, leading to increased expression of these chemokines [48]. The rapid and robust binding of NF-κB to the *CXCL14* promoter is consistent with the idea that viral recognition triggers canonical NF-κB pathways in epithelial cells, which in turn drive early chemokine production. Zhang et al. found that NF-κB was involved in the IL-1β-induced upregulation of CCL3 and CCL4 expression in human primary chondrocytes, particularly at 4 to 8 hours of exposure [49].

Additionally, Liu et al. found that HSV-1 infection of human corneal epithelial cells led to increased NF-κB levels [38]. Our findings position *CXCL14* among the NF-κB–responsive genes upregulated during the initial innate response to HSV-2. Liu et al. suggest a role for the US2 Gene product of HSV-2 (virion protein US2) in inducing NF-κB signaling. They found that US2 interacted with TGF-β-activated kinase 1 (TAK1) to activate the IκB kinase (IKK) complex. This subsequently induced IκB phosphorylation and degradation, leading to NF-κB translocation from the cytoplasm to the nucleus and, subsequently, the production of cytokines and chemokines [50]. This mechanism may explain our observation of the progressive accumulation of NF-κB in the nuclei of HSV-2–infected VECs over time. Once in the nucleus, NF-κB likely binds to the promoters of chemokines, such as CXCL14, thereby contributing to their upregulation during infection.

Our ChIP data indicate that OCT-1 is also recruited to the host *CXCL14* promoter shortly after infection. This novel observation raises interesting questions about OCT-1’s role: it may be indirectly drawn to the *CXCL14* promoter *via* virus-activated co-factors, or OCT-1 itself might recognize consensus sites in the CXCL14 regulatory region under stress conditions. These findings align with prior evidence that HSV-2 can co-opt host OCT-1 through the viral tegument protein VP16. Upon entry, VP16 forms a complex with host HCF-1 and OCT-1, which then binds specific DNA motifs in immediate-early (IE) viral gene promoters and recruits chromatin-modifying enzymes to activate transcription [51]. While this mechanism is classically described for viral gene promoters, our data raise the possibility that OCT-1, possibly in concert with VP16 and associated co-activators, could target host promoters such as *CXCL14*.

CXCL14 can influence T-cell immunity indirectly by recruiting/activating DCs and enhancing DC-mediated T-cell responses; in some settings it may also promote regulatory T-cell activation [52]. Our extensive pathway analysis revealed T-cell activation, chemokine signaling, NF-kB, and chemokine receptor-like pathways to be significantly upregulated among ASYMP human participants with genital herpes. Interestingly, we found the chemotaxis pathway to be upregulated among ASYMP participants. Chemokine-mediated recruitment is important for HSV control, as CXCL9/CXCL10/CXCR3 signaling mobilizes HSV-specific cytotoxic T lymphocytes (CTLs), NK cells, and memory CD8^+^ T cells during genital/recurrent HSV infection; however, chemokine-driven inflammatory pathways can also influence or worsen HSV-associated pathology, including herpes stromal keratitis and severe HSV-2 inflammatory disease [21, 53, 54]. CXCL14 itself has been shown to recruit/affect immune cells relevant to antiviral defense, including activated NK cells and DC lineage cells, but direct evidence that CXCL14 specifically worsens HSV-1 or HSV-2 disease is limited [52, 55, 56]. Another prominent pathway that we found to be upregulated among ASYMP participants is RANTES AND IP-10 pathways. During HSV stimulation, plasmacytoid DCs produce CCL5/RANTES along with other chemokines and promote migration of T cells and NK cells; in genital HSV-2 infection, the CCL5 receptor CCR5 contributes to NK-cell mobilization and antiviral resistance [22, 57]. Other major immunological pathways that we found to be downregulated among ASYMP participants with genital herpes were NK-cell cytotoxicity, Negative regulation of T-cell activation, MCP-1, and T-cell apoptosis pathways. Among these, the MCP-1 pathway is well known for its role in monocyte recruitment, enhancing the inflammatory response during HSV infection [58, 59]. Further, we found the downregulation of the TGF-β pathway among ASYMP participants with herpes. The TGF-β pathway is known to modulate immune responses and can influence the severity of herpes lesions. TGF-β signaling can modulate HSV disease severity by suppressing antiviral inflammatory responses and promoting regulatory T-cell-mediated control of immunopathology; experimentally, TGF-β1 mucosal gene transfer reduced HSV-induced ocular lesion severity, and TGF-β-generated antigen-specific Foxp3+ Tregs controlled HSV-induced ocular immunoinflammatory lesions. CXCL14 may indirectly connect to this regulatory axis because it has been shown to promote immature dendritic-cell accumulation and Treg activation in inflammatory tissue [41, 60, 61].

Integrating our findings with the broader literature reveals that CXCL14 is a critical immunomodulator at epithelial barriers, playing context-dependent roles. In the context of an acute genital HSV-2 infection, CXCL14 acts as a pro-immune, protective chemokine that rapidly recruits innate and adaptive effector cells to control viral replication. This is supported by the association of high CXCL14 levels with ASYMP infection (ie, effective immune containment) and the severe deficits in immune defense observed in *CXCL14(–/–)* mice. The importance of CXCL14 in antiviral defense is further underscored by the fact that some pathogens actively target the CXCL14 pathway. For example, the human papillomavirus (HPV) oncoprotein E7 causes epigenetic silencing of CXCL14 in infected epithelial cells, allowing HPV-driven tumors to evade immune surveillance. Restoring CXCL14 in an HPV^+^ cancer model resulted in increased CD8^+^ T-cell infiltration and tumor suppression [42]. This parallels our observation that, in the absence of CXCL14, HSV-2 proliferates unchecked, suggesting that viruses such as HSV and HPV have significant benefits when CXCL14 is absent or suppressed.

**Figure 9.**
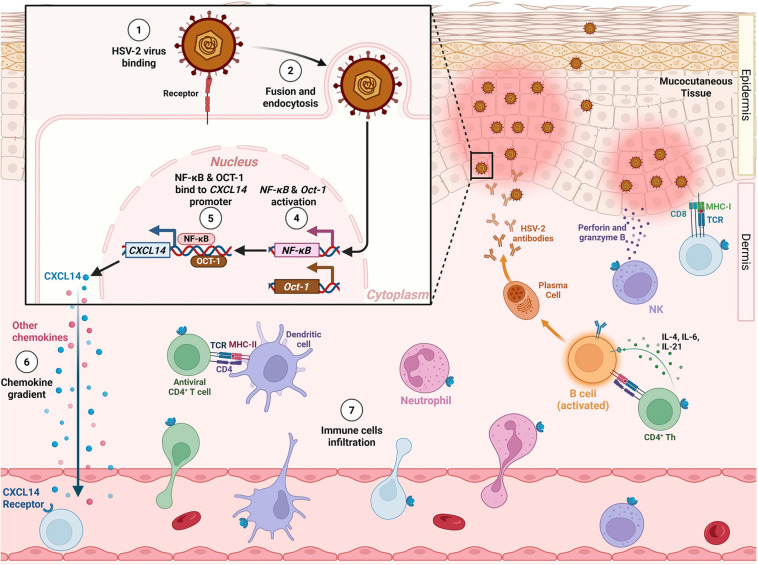
**Proposed mechanism of NF-κB– and OCT-1–mediated regulation of CXCL14 mucosal chemokine during HSV-2 infection and its role in mucosal immune defense.** Following HSV-2 entry into VECs via host cell receptor binding (1) and fusion/endocytosis (2), viral recognition starts activation of NF-κB and OCT-1 (4). These transcription factors translocate to the nucleus and bind the CXCL14 promoter (5), thereby upregulating CXCL14 expression and promoting its secretion. CXCL14, together with other chemokines (6), forms a chemokine gradient that facilitates the infiltration of diverse immune cells to the site of infection (7), including dendritic cells, NK cells, neutrophils, and CD4^+^ and CD8^+^ T cells. These recruited cells mediate antiviral activity via cytotoxic mechanisms (eg, perforin, granzyme B), cytokine release (eg, IL-4, IL-6, IL-21), and antibody production, collectively contributing to effective control of HSV-2 replication and spread within mucocutaneous tissues. Created with BioRender.com.

In summary, as illustrated in Figure 9, our study establishes CXCL14 as a critical chemokine for protective immunity against genital HSV-2. CXCL14 serves as an early alarm and recruitment signal in the VM, drawing in NK cells, CD4^+^ T_EM_ cells, and CD8^+^ T_EM_ cells, as well as other leukocytes, to contain the infection. In the absence of CXCL14, this immune orchestration fails, resulting in insufficient T-cell responses, increased viral replication, and severe disease. These findings enrich our understanding of mucosal immunology by highlighting a non-redundant role for a homeostatic chemokine in acute antiviral defense. Given that mucosal surfaces rely on rapid innate triggers to summon adaptive effectors, CXCL14 appears to be a key link between epithelial sensing of HSV-2 and the deployment of antiviral immunity.

From a clinical and translational perspective, our results suggest that enhancing CXCL14-mediated pathways could improve resistance to HSV-2 and other mucosal pathogens. Traditional approaches, including inactivated “killed” virus, live-attenuated, replication-defective, and subunit glycoprotein vaccines, have largely failed to prevent recurrent HSV-2 disease [62]. For example, a glycoprotein D subunit vaccine with adjuvant (aluminum salt) induced neutralizing antibodies but provided only modest and inconsistent protection in clinical trials. These challenges underscore the need for a new strategy that elicits robust T-cell immunity rather than relying solely on antibodies. Researchers have observed that individuals with better control of genital herpes have robust HSV-2-specific CD4^+^ and CD8^+^ T cells residing in the VM (the site of recurrent lesions) and their dorsal root ganglia (DRG, the site of viral latency) [63]. An ideal therapeutic vaccine should therefore induce tissue-resident memory T cells (T_RM_) in both the central neuronal immunity of the DRG and the peripheral epithelial immunity of the VM. However, memory T cells do not circulate freely into immune-restrictive sites, such as the VM, skin, or lung airways, at steady state; instead, their trafficking is governed by chemokine gradients induced by local infection or inflammation [64]. Chemokines such as CXCL9 and CXCL10, which act through CXCR3, and mucosal chemokines such as CXCL17, CXCL14, and CCL28 have been shown to recruit and retain functional memory T cells within mucosal tissues [39]. Building on this principle, the Prime/Pull/Keep (PPK) strategy represents this paradigm shift: first, prime the host with key HSV antigens to generate a broad T cell response; then, pull those T cells into infected tissues using chemokine cues; and finally, keep them on site for long-term protection. In HSV-2-infected guinea pigs, we demonstrated that intravaginal administration of a neurotropic AAV8 vector encoding CXCL11 significantly enhanced the infiltration of functional CXCR3^+^ CD4^+^ and CD8^+^ T_RM_ and T_EM_ cells in both the DRG and VM, resulting in a substantial reduction in viral shedding and lesion recurrence [65]. Further incorporation of mucosal chemokines, such as CCL28 and CXCL17, has demonstrated additive benefits, facilitating the long-term retention of CCR10^+^ and CXCR8^+^ memory T cells within the VM [25, 37]. By establishing sustained antiviral T-cell immunity in both central and peripheral target tissues, the PPK strategy represents a promising paradigm shift in the development of effective immunotherapeutics for genital herpes.

## MATERIALS AND METHODS

### Mice

Female CXCL14 knockout (*CXCL14(–/–)*) mice on the C57BL/6 background, 6-8 weeks old, were purchased from Taconic Biosciences (GEM-NTMCK-220701-AAE-04 [CY1038, CXCL14]). Female C57BL/6 (B6) wild-type (WT) mice (6–8 weeks old) were purchased from the Jackson Laboratory. Primers used to genotype the *CXCL14* mutant allele are listed in Table 1. The mice were maintained and bred under specific pathogen-free conditions with a 12-hour light/dark cycle and access to food and water (*ad libitum*). Animal studies conformed to the Guide for the Care and Use of Laboratory Animals published by the U.S. National Institute of Health. All procedures were approved by the Institutional Animal Care and Use Committee (IACUC) of the University of California, Irvine (protocol #22-086) and conducted in accordance with the NIH guidelines.

**Table 1.** List of Primer Sequences Used in this Study

Target gene	Forward sequence (5′ – 3′)	Reverse sequence (5′ – 3′)
*CXCL14* genotyping		
*CXCL14*	GTTCCAGGATGCCTAGGTGG	AGAGGGTAGCGGATGTGAGT
HSV-2 quantification		
*Us9*	GGCAGAAGCCTACTACTCGGAAA	CCATGCGCACGAGGAAGT
Gene expression in primary human vaginal epithelial cells		
*CXCL14*	ATGAAGCCAAAGTACCCGCA	CCAGGCGTTGTACCACTTGA
*CXCL17*	CAGTCTTAGCCTGTGCCCTC	GGCAGACCCCATTTGAAGGA
*CCL28*	TGCACGGAGGTTTCACATCA	AGGATGACAGCAGCCAAGTC
*CCL25*	TCCACCAGGTGTGTGCAGATG	GCCACCGATATCCACTGGG
*NF-κB*	GTGGGGACTACGACCTGAATG	GGGGCACGATTGTCAAAGATG
*OCT-1*	TCAGCCCATACAGATCGCAC	TGGCTGCAAATTGGTGGTTG
*GAPDH*	GACAGTCAGCCGCATCTTCT	GCGCCCAATACGACCAAATC
Gene expression in mice		
*CXCL14*	GAGTCACCGAGTGGTTCTGC	GTTCTCGGTTTCAAGCACGC
*CXCL17*	TGTGATCACGTCAAGGGCAG	CTGGAGGGTCTTTGCGACTT
*CCL28*	CCTTCCAGAAATCCCCACCT	CCTGTAAGTGGAGCGACTGTT
*CCL25*	CCAAGGTGCCTTTGAAGACT	TCCTCCAGCTGGTGGTTACT
*GAPDH*	TGAGCCTCCTCCAATTCAACC	AATCCGTTCACACCGACCTT
ChIP-qPCR		
*CXCL14*_P1	AGAGAAAGCCGAGCAGAGC	GAGCAGGGACATGGGGAG
*CXCL14*_P2	AGGTCTCCTCCCCTCACC	CGAGCTCATTAATATGCAGAACC
*CXCL14*_P3	TTAAAAGAGGCCAGGGCGG	TCTGCTCGGCTTTCTCTGC
*CXCL14*_P4	TGGTTACCACTGTCATCGCT	TTAAAGTGGGTCAGTGGCCT
*CXCL14*_P5	CAGAAAAGCAGTGCCCTTCA	CTACCCTCTCGCTCACACAA

### Genital HSV-2 Infection

First, mice were injected subcutaneously (s.c.) with 2 mg progesterone (Depo-Provera) to synchronize the ovarian cycle and increase susceptibility to herpes infection. After Depo-Provera treatment, mice were anesthetized and inoculated intravaginally with 20 µL of HSV-2 strain MS (5 × 10^5^ PFU/mL) per mouse. Vaginal swabs were collected on days 2, 4, 6, and 9 p.i. using type 1 Dacron swabs (Spectrum Laboratories, Los Angeles, CA) (Figure 3A), transferred to a 2 mL sterile cryogenic vial containing 1 mL of culture medium, and stored at −80 °C. On day 14 p.i., mice were euthanized, and immune cells from the VM were used for flow cytometry analysis.

### Monitoring of Genital Herpes Infection and Disease Scoring in Mice

Viral DNA was extracted from swabs using Quick-DNA Viral Kit (Thomas Scientific, D3016), and HSV-2 detection and quantification were performed by quantitative PCR (qPCR) using the primers listed in Table 1. The HSV-2 DNA copy number was determined using purified HSV-2 DNA (Advanced Biotechnologies) and calculated based on a standard curve generated from CT values. Mice were scored daily from day 1 to day 9 p.i. for pathological symptoms. The severity of genital herpetic lesions was scored on a scale of 0−4, where 0 = no disease, 1 = swelling of external vagina; 2 = swelling and redness of external vagina, 3 = severe swelling and redness of vagina and surrounding tissue and hair loss in the genital area, 4 = ulceration and hair loss in the genital and surrounding tissue.

### Human Study Population

In a 15-year period spanning from January 2003 to October 2018, we screened 925 individuals for HSV-1 and HSV-2 seropositivity. A total of 587 participants were White, 338 were nonwhite (African, Asian, Hispanic, and other), 467 were female, and 458 were male. Among this sample, a cohort of 308 immunocompetent individuals, ranging from aged 21 to 69 years (median, 45 years), were seropositive for HSV-2. All patients were negative for HIV and hepatitis B virus (HBV), with no history of immunodeficiency. A total of 278 patients were healthy and defined as ASYMP. These patients never had any herpes disease (genital, orofacial, dermal, or ocular) based on self-reporting and clinical examination. Even a single episode of any herpetic disease excluded the individual from this group. The remaining 30 patients were defined as HSV-seropositive SYMP individuals who suffered from frequent and severe recurrent genital lesions. Patients were also excluded if they (i) had an active genital (or elsewhere) herpetic lesion or had one within the past 30 days, (ii) were pregnant or breastfeeding, or (iii) were on acyclovir and other related antiviral drugs or any other immunosuppressive drugs at the time of blood draw. SYMP and ASYMP groups were matched for age, sex, serological status, and race. Among this large cohort, 4 SYMP and 4 ASYMP women were enrolled in the current study for RNA sequencing study to evaluate the differentially expressed genes during genital herpes infection.

All participants were enrolled at the University of California, Irvine, under approved Institutional Review Board-approved protocols (IRB no. 2003-3111 and IRB no. 2009-6963). Written informed consent was received from all participants prior to inclusion in the study.

### HSV-specific Serotyping

The sera collected from random donors were tested for anti-HSV antibodies. ELISA was performed on sterile 96-well flat-bottom microplates coated with the HSV-2 antigen in coating buffer overnight at 4°C. The next day, plates were washed with phosphate-buffered saline (PBS)–1% Tween 20 (PBST) 5 times, and nonspecific binding was blocked by incubation with a 5% solution of skimmed milk in PBS (200 μl/well) at 4°C for 1 hour at room temperature (RT). The microplates were washed 3 times with PBS-Tween and incubated with various sera at 37°C for 2 hours. Following 5 washes, biotinylated rabbit anti-human IgG, diluted 1: 20,000 with PBST, was incubated at 37°C for 2 hours. After 5 washes, streptavidin-peroxidase was added at a 1: 5,000 dilution and incubated for 30 minutes at RT. After 5 additional washes, the color was developed by adding 100 μl of tetramethylbenzidene (TMB) substrate. The mixture was incubated for 5 to 15 minutes at RT in the dark. The reaction was terminated by adding 1 M H_2_SO_4_. The absorbance was measured at 450 nm.

### Peripheral Blood Mononuclear Cell Isolation

Individuals (negative for HIV and HBV and with or without any HSV infection history) were recruited at the UC Irvine Institute for Clinical and Translational Science (ICTS). One hundred milliliters of blood was drawn into yellow-top Vacutainer tubes (Becton, Dickinson). Peripheral blood mononuclear cells (PBMC) were isolated by gradient centrifugation using leukocyte separation medium (Life Sciences, Tewksbury, MA). The cells were then washed in PBS and suspended in complete culture medium consisting of RPMI 1640, 10% fetal bovine serum (FBS; Bio-Products) supplemented with 1× penicillin–streptomycin–l-glutamine, 1× sodium pyruvate, 1× nonessential amino acids, and 50 μM 2-mercaptoethanol (Life Technologies).

### Bulk RNA Sequencing on Sorted CD8^+^ T cells

We performed gene expression profiling analysis using RNA-Seq on 8 human samples. All samples were female patients with herpes categorized into SYMP (n = 4), and ASYMP (n = 4). RNA was isolated from the sorted CD8^+^ T cells using the Direct-zol RNA MiniPrep (Zymo Research) according to the manufacturer’s instructions. RNA concentration and integrity were determined using the Agilent 2100 Bioanalyzer. Sequencing libraries were constructed using TruSeq Stranded Total RNA Sample Preparation Kit (Illumina). Briefly, rRNA was first depleted using the RiboGone rRNA removal kit (Clonetech Laboratories) before the RNA was fragmented, converted to double-stranded cDNA and ligated to adapters, amplified by PCR, and selected by size exclusion. Following quality control for size, quality, and concentrations, libraries were multiplexed and sequenced to single-end 100-bp sequencing using the Illumina HiSeq 4000 platform.

### Differential Gene Expression Analysis

Differentially expressed genes (DEGs) were analyzed by using integrated Differential Expression and Pathway analysis tools. Integrated Differential Expression and Pathway analysis seamlessly connect 63 R/Bioconductor packages, 2 web services, and comprehensive annotation and pathway databases for *homo sapiens* and other species. The expression matrix of DEGs was filtered and converted to Ensemble gene identifiers, and the preprocessed data were used for exploratory data analysis, including *k*-means clustering and hierarchical clustering. The pairwise comparison of SYMP and ASYMP groups was performed using the DESeq2 package with a threshold of false discovery rate < 0.5. and fold change >1.5. Moreover, a hierarchical clustering tree and network of enriched GO/KEGG terms were constructed to visualize the potential relationship. Gene Set Enrichment Analysis (GSEA) method was performed to investigate the related signal pathways activated among SYMP and ASYMP groups. The Parametric Gene Set Enrichment Analysis (PSGEA) method was applied based on data curated in Gene Ontology and KEGG. The pathway significance cutoff with a false discovery date (FDR) ≥ 0.2 was applied.

### Immunofluorescence (IF) Staining of Tissue Sections

VM tissues from HSV-2-infected mice were fixed in 10% neutral-buffered formalin, embedded in paraffin, and sectioned at a thickness of 8 µm. Tissue sections were dewaxed in xylene, rehydrated through a descending ethanol scale, and underwent antigen retrieval (Vector Labs, #h-3301). Staining was performed to visualize mucosal chemokines, CD4^+^ and CD8^+^ T-cell infiltration, and HSV-2 antigen in the infected VM, as previously described [66]. The primary and secondary antibodies used are listed in Table 2. The slides were mounted with ProLong Diamond Antifade Mountant (Life Technologies, #P36970), and Images were acquired using a Keyence BZ-X810 fluorescence microscope. CD4^+^ T cell infiltration was quantified using ImageJ on immunofluorescence images. For each experimental group, 3 mice were analyzed, and 3 non-overlapping fields of view per mouse were quantified. All images were acquired using identical microscope settings. CD4^+^ cells were identified based on a specific fluorescence signal and quantified within a defined tissue area for each field. The values from the 3 fields were averaged per mouse, and mouse-level means were used for statistical analysis.

**Table 2.** List of Antibodies Used in This Study

Target	Host Species	Dilution	Application (in this study)	Company	Ref. number
Primary antibodies					
CXCL14	Rabbit	1:1000	ICC, IHC	GeneTex	#GTX108431
CXCL17	Rat	1:250	IHC	Invitrogen	#510614
CCL25	Rat	1:250	IHC	GeneTex	#GTX53268
CCL28	Rabbit	1:500	IHC	abcam	#ab231557
CD4	Rat	1:100	IHC	Invitrogen	#14-9766-82
CD8a	Rat	1:100	IHC	Invitrogen	#14-0808-82
HSV-2	Mouse	1:200	IHC	GeneTex	#GTX40663
NF-κB p65	Rabbit	5 µg	ChIP, ICC	Invitrogen	#51-0500
Oct-1	Rabbit	5 µg	ChIP	abcam	#ab308043
c-Jun (AP-1)	Rabbit	1:50	ChIP	Invitrogen	#MA5-15172
Blimp-1	Mouse	5 µg	ChIP	Invitrogen	#MA1-16874
ZNF683 (Hobit)	Rabbit	5 µg	ChIP	Invitrogen	#PA5-54902
Secondary antibodies					
Target (Anti-)	Host Species	Dilution	Conjugate	Company	Ref. number
Rabbit	Goat	1:500	Alexa Fluor™ 488	Invitrogen	#A-11012
Mouse	Goat	1:500	Alexa Fluor™ 488	Invitrogen	#A-11001
Rat	Donkey	1:250	Alexa Fluor™ 488	Invitrogen	#A-21208
Flow cytometry antibodies					
Target	Clone	Dilution	Fluorochrome	Company	Ref. number
CD4	GK1.5	1:100	BV421	BioLegend	#10043
CD8	53-6.7	1:100	APC	BioLegend	#100714
CD11c	N418	1:100	PerCP	BioLegend	#117326
CD19	6D5	1:100	BV785	BioLegend	#115543
CD44	IM7	1:100	Alexa Fluor-700	BioLegend	#103026
CD62L	MEL-14	1:100	BV421	BioLegend	#562910
CD68	FA-11	1:100	BV421	BioLegend	#137017
MHC-II	M5/114.15.2	1:100	APC	BioLegend	#107614
B220	RA3-6B2	1:100	BV421	BioLegend	#103251
NK1.1	PK136	1:100	PE	BioLegend	#108707
Ly6G	1A8	1:100	PE/Cy7	BioLegend	#127618
F4/80	QA17A29	1:100	FITC	BioLegend	#157310
IFN-γ	XMG1.2	1:100	PE/Cy7	BioLegend	#505826
TNF-α	MP6-XT22	1:100	APC-Cy7	BioLegend	#506308
GzmB	QA16A02	1:100	APC	BioLegend	#372204

### Gene Expression Analysis

VM tissues were harvested, placed in 1 mL RNAlater Stabilization Solution (Fisher Scientific, AM7024), and then stored at −80 °C until processing. Approximately 50 mg of tissue was homogenized at 30 Hz for 1 minute using a Tissue Lyser II (QIAGEN). Total RNA was extracted using the miRNeasy Mini Kit (Qiagen #217004), and RNA concentrations were measured using a NanoDrop 1000 (ThermoFisher Scientific). Complementary DNA (cDNA) was synthesized using the High-Capacity cDNA Reverse Transcription Kit (ThermoFisher Scientific, #4368814). qPCR was performed to assess the expression of mucosal chemokines using PowerUp SYBR Green Master Mix (ThermoFisher Scientific, #A25741) on a QuantStudio 5 Real-Time PCR System (ThermoFisher Scientific). Primers sequences used in the qPCR are listed in Table 1. Target gene expression levels were normalized to *GAPDH* mRNA. Fold changes in gene expression were relative to average expression in uninfected control samples and calculated using the 2^−ΔΔCT^ method.

Flow cytometry single-cell suspensions were prepared from the VM of each mouse by enzymatic digestion using collagenase (8 mg/mL) for 1hour. Following digestion, the cells were filtered through a 70-μm strainer to obtain a uniform single-cell suspension, which was then processed for flow cytometry staining. The antibodies used for surface staining are listed in Table 2. Surface staining was performed by incubating 1 × 10^6^ cells in PBS supplemented with 1% fetal bovine serum (FBS) and 0.1% sodium azide, along with the antibody mix, for 45 minutes at 4 °C. After staining, cells were washed 3 times in FACS buffer (PBS with 1% FBS and 0.1% sodium azide) and fixed in 2% paraformaldehyde (Sigma-Aldrich, St. Louis, MO). Quantification of DCs (CD-11c^+^MHC-II^+^), total T cells (CD8^+^ and CD4^+^), and memory T cell subsets (CD8^+^CD44^+^ and CD4^+^CD44^+^), including central memory (T_CM_: CD62L^+^) and effector memory (T_EM_: CD62L^–^) cells, was compared using flow cytometry in vaginal cell suspensions of age-matched CXCL14(–/–) mice vs. WT controls.

For intracellular staining, cells were treated with Cytofix/Cytoperm solution (BD Biosciences) following surface staining and incubated for 45 minutes at 4 °C with antibodies against IFN-γ, TNF-α, and Granzyme B (Table 2). Cells were then washed with FACS buffer and fixed again with 2% paraformaldehyde. A total of 100,000 lymphocyte-gated PBMC events were acquired on a BD Fortessa X20 flow cytometer (Becton Dickinson). Data were analyzed using FlowJo software version 10.10.0 (Becton Dickinson). Fluorescence-minus-one (FMO) and unstained controls were included to guide the gating strategy.

### Cell Culture and Virus Preparation

Primary human VECs (PCS-480-010) were obtained from the American Type Culture Collection (ATCC) and cultured in vaginal epithelial cell basal medium (ATCC PCS-480-030) supplemented with vaginal epithelial cell growth kit (ATCC PCS-480-040) at 37 °C in a humidified atmosphere containing 5% CO_2_. Cells were grown in monolayers, and the medium was refreshed every 48h. For passaging, cells were detached using a trypsin-EDTA solution (Corning #25-052-CI) and neutralized with trypsin neutralizing solution (ATCC PCS-999-004). Cells were counted using a hemocytometer and seeded at a density of 5000 cells/cm^2^.

For infection experiments, primary human VECs were plated in 12-well plates at 5000 cells per well. After 48 hours, cells were infected with the HSV-2 strain MS at an MOI of 10 for 1 hour at 37°C in 5% CO₂. Subsequently, the unabsorbed virus was removed, and fresh vaginal epithelial cell basal medium was added. Cells were collected at various time points (0, 2, 4, 8, 12, 24, 36, 48, 72, 96, and 120 hours) for gene expression analysis, (0, 12, 24, 36, 48, 72, 96, and 120 hours) for viral DNA quantification, and at selected time points (0, 12, 24, and 36 hours) for Immunocytochemistry (ICC). Viral DNA extraction, HSV-2 qPCR quantification, and gene expression analysis in human VECs were performed using the same procedures described for tissue samples.

### Immunocytochemistry (ICC) by Immunofluorescence

Primary human VECs grown on Millicell EZ slides (Millipore, #PEZGS0416) were infected with HSV-2 strain MS as described above. Six hours before fixation, 1 µL of BD GolgiPlug™ protein transport inhibitor (Fisher Scientific, #BDB555029) was added to each well to block chemokine transport outside the infected cells. At the indicated time points (12, 24, and 36h p.i.), cells were washed once with PBS and then fixed using 4% paraformaldehyde (BioWorld, #30450002) for 10 min at room temperature. The fixed cells were washed three times with PBS, permeabilized with 0.2% Triton X-100 (Sigma-Aldrich, #T9284) in PBS for 10 minutes at room temperature, and then blocked with blocking buffer (3% bovine serum albumin in PBS) for 1 hour at room temperature.

The cells were incubated with the primary antibody (Table 2) diluted in blocking buffer at 4° C overnight. The following day, the cells were washed four times with PBS and incubated with fluorescently labeled secondary antibodies diluted in blocking buffer for two hours at room temperature. The cells were then washed four times in PBS and incubated with DAPI diluted in PBS for 15 min at room temperature. Finally, cells were washed three times in PBS and mounted with ProLong Diamond Antifade Mountant (Life Technologies, #P36970). Imaging was performed using a Keyence BZ-X810 fluorescence microscope.

### Chromatin Immunoprecipitation (ChIP)

The interaction between TFs (NF-κB, OCT-1, AP-1, and Blimp-1) and the *CXCL14* promoter was performed using the MAGnify Chromatin Immunoprecipitation System (ThermoFisher Scientific, #492024) according to the manufacturer’s protocol. Briefly, primary human VECs were infected with HSV-2 (MOI = 10) for 2, 4, 12, 24, and 72 hours. Both infected and non-infected cells were crosslinked with formaldehyde (1% final concentration) for 10 minutes, followed by quenching with 1.25 M glycine for 5 minutes, all at room temperature. Cells were then washed, collected, pelleted by centrifugation, and lysed with lysis buffer supplemented with protease inhibitor. DNA was sheared by ultrasonication to generate fragments of 300-500 base pairs. Soluble chromatin was aliquoted and immunoprecipitated using magnetic beads conjugated with antibodies against NF-κB, OCT-1, AP-1, and Blimp-1 (Table 2).

Immunoprecipitated complexes were processed following the manufacturer’s instructions. DNA was purified from ChIP and input samples, and qPCR was performed at the *CXCL14* promoter region. Enrichment of target DNA regions was quantified and compared with that of an isotype-matched IgG control. Primer sequences used for analysis are listed in Table 1.

### Statistical Analysis

Mean is expressed as mean ± standard deviation. Statistical analysis was conducted using GraphPad PRISM 10.5.0. The unpaired Student’s *t-*test with Welch’s correction and one-way ANOVA were used for statistical analysis. Statistical significance was established at *P* < 0.05.

## SUPPLEMENTARY DATA

Supplementary materials are available at the *Pathogens and Immunity* website. Supplementary data may be provided by the authors to benefit the reader. Supplementary data are not copyedited and are the sole responsibility of the authors. Questions or comments related to supplementary materials should be addressed to the corresponding author.
