# Supplementary material for: NF-κB/OCT−1 Mediated Upregulation of CXCL14 Chemokine Mobilizes Mucosal Effector Memory CD44+CD62L-CD4+ and CD8+ TEM Cells, and NK Cells Associated with Protection Against Genital Herpes
Source: Pathog Immun. 2026 Jun 3;11(1):191–222. doi: 10.20411/pai.v11i1.879 (PMC13410923; doi:10.20411/pai.v11i1.879)
Supplement: Supplementary Figures [file pai-11-191-s01.pdf]

PATHOGENS AND IMMUNITY
IN ASSOCIATION WITH CASE WESTERN RESERVE UNIVERSITY AND THE RICHARD J. FASENMYER FOUNDATION

Supplementary Figures

Lekbach et al. Supplementary Figure S1

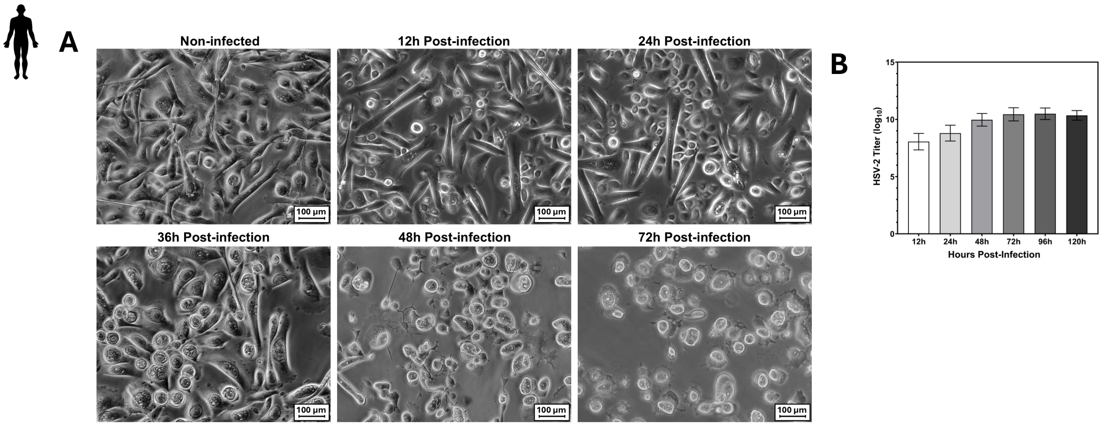

Supplementary Figure 1. HSV-2 infection induces cytopathic effects and viral replication in human vaginal epithelial cells. (A) Phase-contrast microscopy images showing morphological changes in human vaginal epithelial cells at various time points following HSV-2 infection (12, 24, 36, 48, and 72h p.i.). Non-infected cells display a healthy monolayer with typical epithelial morphology. Progressive cytopathic effects, including cell rounding, detachment, and lysis, are observed starting at 24 hours post-infection (p.i.) and become more pronounced through 72 hours p.i. Scale bars: 100 µm. (B) Quantification of HSV-2 replication over time by viral genomic DNA level measurements (log10) at 12, 24, 48, 72, 96, and 120 hours p.i. HSV-2 genomic DNA levels increase steadily, reaching peak levels at later time points, confirming productive viral replication in infected human vaginal epithelial cultures. Bars show the mean ± SD.

Lekbach Y, Prakash S, Vahed H, Quadiri A, Rahman A, El Fatimi EH, Dorotta JC, Suoth BS, Maurya C, Garcia A, Park G,, BenMohamed L. NF-κB/OCT-1 Mediated Upregulation of CXCL14 Chemokine Mobilizes Mucosal Effector Memory CD44+CD62L-CD4+ and CD8+ TEM Cells, and NK Cells Associated with Protection Against Genital Herpes. *Pathogens and Immunity*. 2026;11(1):191–222. doi: 10.20411/pai.v11i1.879

PATHOGENS AND IMMUNITY
IN ASSOCIATION WITH CASE WESTERN RESERVE UNIVERSITY AND THE RICHARD J. FASENMYER FOUNDATION

Lekbach et al. Supplementary Figure S2

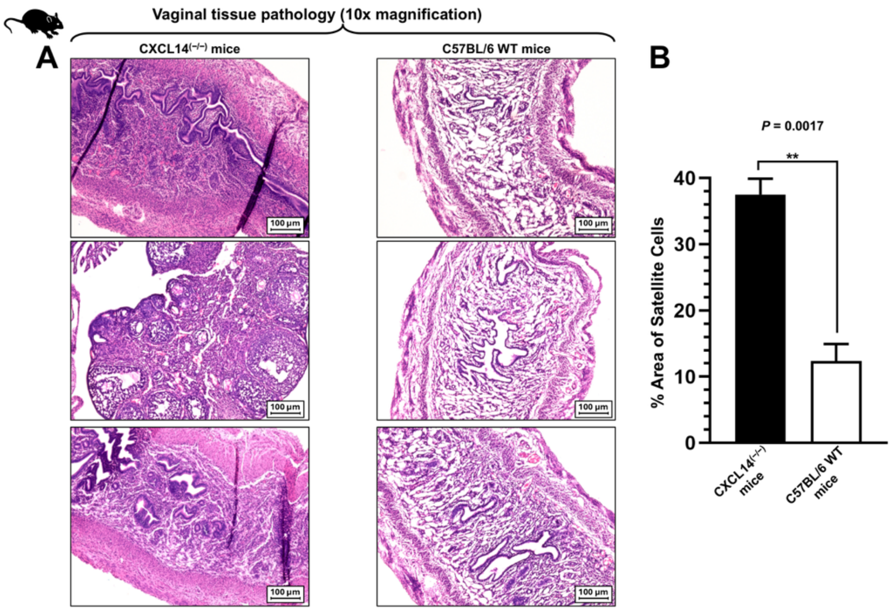

Supplementary Figure 2. CXCL14 deficiency enhances HSV-2-mediated pathology in the vaginal tissue. (A) Representative Hematoxylin and eosin (H&E) staining of vaginal tissues at 10x magnification show higher degree of epithelial inflammation in *CXCL14(⁻/⁻)* mice compared to WT mice. All images are shown at a 100 μm scale. (B) Quantitative analyses were performed using ImageJ software. Bar graphs represent mean ± SD, n = 10 mice/group. Statistical significance was determined using an unpaired *t*-test ($P < 0.05$).

Lekbach Y, Prakash S, Vahed H, Quadiri A, Rahman A, El Fatimi EH, Dorotta JC, Suoth BS, Maurya C, Garcia A, Park G,, BenMohamed L. NF-κB/OCT-1 Mediated Upregulation of CXCL14 Chemokine Mobilizes Mucosal Effector Memory CD44+CD62L-CD4+ and CD8+ TEM Cells, and NK Cells Associated with Protection Against Genital Herpes. *Pathogens and Immunity*. 2026;11(1):191–222. doi: 10.20411/pai.v11i1.879
